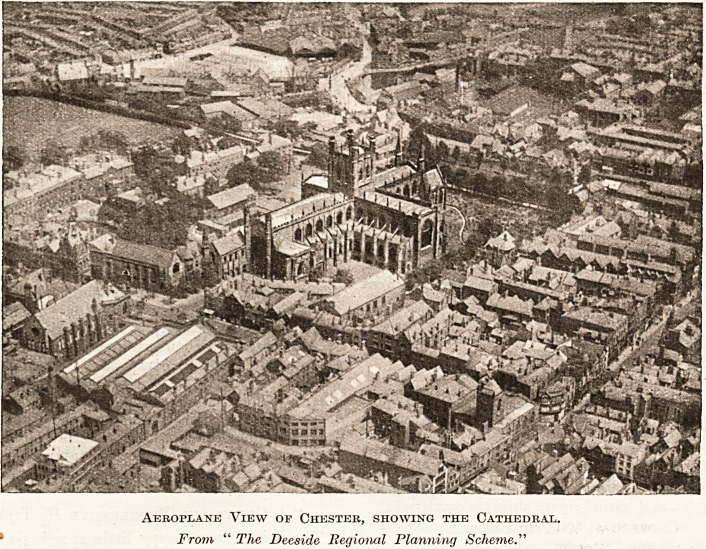# Zoning the Worker

**Published:** 1923-08

**Authors:** 


### ZONING THE WORKER.

### A DEESIDE REGIONAL PLANNING SCHEME.

IN our issue for February we published a brief review of the Doncaster Regional Planning Scheme—a pioneer document of the utmost value drawn up at the suggestion of the Minister of Health. The Doncaster Report has now been followed by the publication, through the University Press of Liverpool and Messrs. Hodder and Stoughton (7s. 6d. net), of the Deeside Regional Planning Scheme, which deserves to be studied with close attention by all who are concerned for the preservation of the beauties of England and for the good health of her people.

### A Region of Contrasts.

The region to be zoned is one presenting marked contrasts in its residential area, possessing picturesque and healthy towns and villages as well as "conglomerations of houses that hardly merit the name of communities at present save of the worst features of nineteenth century so-called prosperity." Much of the housing in the region is of unrelieved ugliness. "It is not a case of the East End of London, or the slums of Manchester or Liverpool, where square miles of dreariness block out the country. Everywhere in this region the country is accessible in several minutes' walk, and one finds that a few rows of cottages with open fields at the end can be as congested and nearly as gloomy as if they were in the heart of a great city." The region itself is a healthy one with a fairly equable climate, and the population generally is sparse except for certain concentrations in the Deeside centres and in Chester itself:—

"It would be extremely valuable if a Regional Housing and Health Survey were to be made by which the real density of housing areas were made evident (not the average density over a whole district including agricultural land). These densities and the heights above ordnance datum could then be equated with death and disease rates for the same place and the necessity for eliminating the unhealthy houses and planning the new ones on better lines and on healthier sites clearly demonstrated."

Aeroplane View of Chester, showing the Cathedral.
*From "The Deeside Regional Planning Scheme."*

### The Worker and His Home.

The Report covers the city of Chester and the county of Flint, and the recommendations naturally require to be supplemented by, as well as embodied in, local town planning schemes:—

"The chief recommendations may be summed up as an attempt to make the home more attractive and healthy and the work place more efficient, taking into account inter-communication between the two, in order that the minimum of time may be wasted in the useless occupation of diurnal journeyings. The time has come when certain areas must be definitely earmarked for certain uses to prevent that unfortunate mixing up of houses and factories which is detrimental to both of them. It is only by having a plan, and an agreed plan, upon which to work, that the old haphazard methods of development can be avoided. Furthermore, it is recognized that this plan cannot be contained in a series of watertight compartments coinciding with the boundaries of Local Authorities, but must be based upon the requirements of a broad geographical unit."

### The Industrial Outlook.

Rich in antiquities—the region possesses the fine old towns of Chester and Flint, the ruins of old Hawarden Castle and of Bassingwerk Abbey, and the mediæval shrine at Holywell—its industrial outlook is at first sight somewhat depressing—" an apparently exhausted coalfield and lead mines that are flooded mean forsaken workings and derelict chimneys." Nevertheless, the presence of coal and of a wealth of lead and zinc in addition to numerous quarries suggests a prosperous future provided increased transport facilities can be obtained :—

" In allocating the principal areas, regard has been had to suitability by reason of natural physical conformation of land and convenience for transport. Equally important is the due separation of residential from factory land and the restriction of houses to areas desirable from the point of view of health amenity. Manufacturing areas group themselves under two headings—unrestricted or heavy trades, limited or light trades, conforming to stringent requirements as to noise, smoke, dust, smell and size. . . . Generally speaking, quarrying and mining are confined to the higher ground, the Halkyn Ridge and its more irregular continuation towards the coast. . . . A new and extremely important residential area is likely to grow up in the neighbourhood of Hawarden. Here the ground rises gently and continuously from the Dee ; and the proximity to the factory area would give that close connection between home and work which is so desirable as a time saver, while the agricultural belt acts as a satisfactory ' buffer ' between houses and factories."

### The " Ribbon " Development.

Further down the Dee an unfortunate type of growth has occurred owing to the natural conformation of the ground :—

" This consists of a long narrow and almost continuous strip following the road : what has been called the ' ribbon ' type of development. Every effort should be made at certain points to group into units this growth, as has already occurred at Flint owing to the placing of the mediæval town at a point where there was a comparatively level patch set back from the river."

### The Recreations of the People.

With reference to recreational areas it is impossible yet to earmark any large tracts :—

" Land zoned for residential use naturally carries with it the implication of play-grounds laid out as development occurs ; and it might be emphasised how important it is for places such as Connah's Quay, Flint, Bagillt, etc., to safeguard early some of the attractive little valleys which are of no use for agriculture or industries. There are also from time to time opportunities of acquiring at almost nominal prices the old houses and parks with which this region abounds, and which are frequently changing hands at low figures, as industrial growth alters the countryside."

### Chester as a Civic Centre.

Chester with its Roman and mediæval remains, its Cathedral and its river, will naturally form the chief civic centre of the region, and " offers one of the most attractive fields for the preparation of a civic survey to be found in this country." Other centres may spring up at Hawarden or Holywell or Flint, and sections of the Report deal in detail with these places. In the mixed areas—restricted factory zones and the neutral zone—where houses and factories may both be found, some limitation of the heights of buildings will be necessary, as also in the purely residential areas in order to discourage lofty tenement buildings.

### A Valuable Scheme.

The Report has been prepared by Professor Patrick Abercrombie, A.R.I.B.A., who was jointly responsible for the admirable Doncaster Report ; Mr. Theodore Fyfe, F.R.I.B.A. ; and Mr. Sydney Kelly, F.S.I. It is excellently illustrated with numerous blocks and maps, and is far from being of merely local interest. The illustration we reproduce is printed by the courteous permission of the publishers. The value of schemes of this kind cannot be overestimated, and their adoption may well cause a real revolution in the life of the industrial worker, making him—what he is not always at present—happy, healthy and well housed.

## Figures and Tables

**Figure f1:**